# Clinician Perspectives on Using Computational Mental Health Insights From Patients’ Social Media Activities: Design and Qualitative Evaluation of a Prototype

**DOI:** 10.2196/25455

**Published:** 2021-11-16

**Authors:** Dong Whi Yoo, Sindhu Kiranmai Ernala, Bahador Saket, Domino Weir, Elizabeth Arenare, Asra F Ali, Anna R Van Meter, Michael L Birnbaum, Gregory D Abowd, Munmun De Choudhury

**Affiliations:** 1 School of Interactive Computing Georgia Institute of Technology Atlanta, GA United States; 2 The Zucker Hillside Hospital Northwell Health Glen Oaks, NY United States; 3 The Feinstein Institutes for Medical Research Manhasset, NY United States; 4 The Donald and Barbara Zucker School of Medicine at Hofstra/Northwell Hempstead, NY United States; 5 College of Engineering Northeastern University Boston, MA United States

**Keywords:** mental health, social media, information technology

## Abstract

**Background:**

Previous studies have suggested that social media data, along with machine learning algorithms, can be used to generate computational mental health insights. These computational insights have the potential to support clinician-patient communication during psychotherapy consultations. However, how clinicians perceive and envision using computational insights during consultations has been underexplored.

**Objective:**

The aim of this study is to understand clinician perspectives regarding computational mental health insights from patients’ social media activities. We focus on the opportunities and challenges of using these insights during psychotherapy consultations.

**Methods:**

We developed a prototype that can analyze consented patients’ Facebook data and visually represent these computational insights. We incorporated the insights into existing clinician-facing assessment tools, the Hamilton Depression Rating Scale and Global Functioning: Social Scale. The design intent is that a clinician will verbally interview a patient (eg, How was your mood in the past week?) while they reviewed relevant insights from the patient’s social media activities (eg, number of depression-indicative posts). Using the prototype, we conducted interviews (n=15) and 3 focus groups (n=13) with mental health clinicians: psychiatrists, clinical psychologists, and licensed clinical social workers. The transcribed qualitative data were analyzed using thematic analysis.

**Results:**

Clinicians reported that the prototype can support clinician-patient collaboration in agenda-setting, communicating symptoms, and navigating patients’ verbal reports. They suggested potential use scenarios, such as reviewing the prototype before consultations and using the prototype when patients missed their consultations. They also speculated potential negative consequences: patients may feel like they are being monitored, which may yield negative effects, and the use of the prototype may increase the workload of clinicians, which is already difficult to manage. Finally, our participants expressed concerns regarding the prototype: they were unsure whether patients’ social media accounts represented their actual behaviors; they wanted to learn how and when the machine learning algorithm can fail to meet their expectations of trust; and they were worried about situations where they could not properly respond to the insights, especially emergency situations outside of clinical settings.

**Conclusions:**

Our findings support the touted potential of computational mental health insights from patients’ social media account data, especially in the context of psychotherapy consultations. However, sociotechnical issues, such as transparent algorithmic information and institutional support, should be addressed in future endeavors to design implementable and sustainable technology.

## Introduction

### Background

Mental health treatment relies heavily on what the patient tells their clinician during in-person consultations. However, issues of retrospective recall bias [1,2], impression management goals [3], and social desirability bias [4] have motivated mental health clinicians to augment patient reports using *collateral information* [5], such as those obtained from patients’ friends and family members. According to the George Engel biopsychosocial model of care [6], such information provides a complementary and adjuvant perspective on the patient’s condition, which the clinician can use to tailor treatment decisions, regulate the quality of care, and support the patient on the road to recovery [5].

The ubiquity and increasing use of digital technology have opened up new opportunities for clinicians to gather complementary sources of collateral information, which can be diverse in scope and gathered in the natural contexts of the patients [7]. Patient-generated health data of patients with irritable bowel syndrome, such as food intake and abdominal pain, have been explored in provider-patient collaboration [8]. The providers saw that self-monitoring data could support provider-patient communication; parallelly, they were also worried about insufficient time to review the data or not having meaningful results from such investments. Kim et al [9] developed DataMD, a clinician-facing patient-generated health data dashboard, by conducting design workshops with clinicians. They found that DataMD helped clinicians to improve counseling skills and facilitated in-depth communication between a clinician and patient.

Among the different types of data sources that can provide collateral information, patients’ social media activities have been investigated in diverse settings such as healthy eating [10] and forensic mental health evaluations [11,12]. Researchers have suggested that social media platforms have emerged as low-cost and unobtrusive means to gather insights about behaviors, mood [13], psychological traits [14], social interactions [15], and even the mental health states of individuals [16,17]. As these platforms provide an unprompted medium through which individuals can voice their feelings and daily experiences, digital traces left behind by people on these platforms provide opportunities for clinicians to gain another layer in their understanding of patients [7,18].

In the wake of these opportunities, clinicians have expressed interest in exploring the use of patients’ social media as clinically relevant information [19]. At the same time, they have been keen to weigh the benefits and drawbacks of doing so [20]. Various studies have suggested ethical guidelines, such as professional boundary management and informed consent, when incorporating social media into clinical settings [21-25]. Even if patients are fully informed, it is unclear how they will share their social activities and to what extent and how the sharing will inform clinicians’ decision-making processes [26,27]. It is also possible that fully informed consented patients may alter their behaviors, which weakens the usefulness of the collateral information from social media data [28]. Moreover, the collateral information derived from patients’ social media should be relevant to the clinical context and provided in a way that clinicians can access their current workflows [29].

Therefore, further research is required to create social media–based technologies that can empower clinician-patient collaboration as collateral information while preventing such technologies from exacerbating ethical concerns. Future technologies need to be able to protect professional boundaries when clinicians and patients collaborate using social technologies. In addition, patients’ privacy must be respected even if the patients have consented to share their social media posts. One of the potential solutions is to computationally translate patients’ social media posts into clinically meaningful insights such as the intensity of certain symptoms [30] and the possibility of relapse [31]. By only showing possibilities or indexes calculated from social media posts, some of the ethical concerns mentioned above can be assuaged; clinicians will not read what the patient posted but will be able to glean important information such as indicators of exacerbation of their symptoms. However, this approach creates other questions: What are the relevant and useful information derived from patients’ social media data? How would clinicians incorporate this information into current work practices? How would new technologies be salient in addressing the ethical concerns of using sensitive personal information in a clinical context?

### Objectives

To examine how collateral information computationally derived from patients’ social media can support or hinder mental health therapy, we developed a clinician-facing prototype that visually represents patients’ social media data. We focused on patients with mood disorders because the collateral information that can be distilled from patients’ social media is relevant to patients with mood disorder [32]. We further left our target condition broad because of the early and exploratory nature of this study.

The prototype was qualitatively evaluated by 15 mental health clinicians. The evaluation study accomplished 2 goals: (1) it helped us understand whether and for what purpose clinicians could incorporate the prototype and social media insights gleaned from patients’ data into their work practices and (2) it revealed concerns and potential harms in its use and adoption in real-world clinical settings. In this study, we present the findings from the user study sessions with mental health clinicians and the implications for future mental health technologies as well as ethical considerations of using patients’ social media data in the mental health context.

## Methods

### Overview

On the basis of the low-fidelity prototypes designed by the research team [29], we developed a prototype with Facebook data of consented patients with mood disorder in treatment at a large health center in the northeast of the United States. The prototype was qualitatively evaluated by clinicians via interviews (n=15) and focus groups (n=13) at this location. The following subsections explain the details of the prototype and the evaluation methods.

### Prototype

#### Overview

As clinicians may be unaccustomed to the concept of computational mental health insights from social media data, we decided to design a prototype that can help clinicians understand this concept and envision its future. The design of our prototype is based on our previous codesign work to understand how computational social media analyses can be visually represented by clinicians [29]. We extended the previous low-fidelity prototypes in 2 ways: first, we used actual patient social media data in the design of the prototype because the insights generated from the actual data and deidentified vignette of the patient can help our clinician participants to evaluate the opportunities of the computational approaches; second, we situated the computational mental health insights as a part of existing clinician-facing assessments because those assessments provide our participants with a familiar base of understanding. A detailed explanation of patient social media data and the design of the prototype are provided below.

#### Patient Facebook Data

In general, this study draws on data from a larger study, some of which have been reported in the studies by Saha et al [30], Birnbaum et al [31], and Ernala et al [33]. In this study, the Facebook archives of a set of clinically diagnosed patients with mood disorder were downloaded following informed consent from the patients and after approval by the institutional review boards of the relevant institutions. From 110 patients who contributed their data following informed consent, we selected an exemplar set of 8 patients with mood disorder, who had the highest activity on Facebook, to build the prototype. Overall, patients had an average of 7143.4 (SD 3209.1) timeline posts and 21,043.6 (SD 16,761.6) messages spanning between 1 and 10 years (mean 6.5, SD 3.6) on Facebook. In particular, the following types of data were used for the specific purposes of our prototype: self-posts and self-comments (posts, comments, and interpersonal messages posted by the patient), including their time of posting, check-ins, friending and cotagging activities, and volume of interpersonal social interactions. In addition to Facebook data, we also accessed their primary diagnosis and hospitalization information (eg, admission and discharge dates) from their medical records.

We used a number of computational analyses on the Facebook data of patients, grounded in the symptomatic and functional impairments associated with mood disorders [29]. To identify posts indicative of depressed mood and suicidal ideation, we used machine learning classifiers (bag-of-words–based 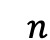-gram models) from prior research [30]. The depression classifier showed an accuracy of 0.82, and the suicidal ideation classifier had an accuracy of 0.91. To capture insomnia, we calculated the number of Facebook posts that were posted during regular sleep hours (between midnight and 5 AM). For diurnal variation in association to mood, we calculated the number of depression-indicative posts (as predicted by the classifier) that were posted at different times of the day (morning, noon, night, and midnight defined between 5 AM and noon, noon and 5 PM, 5 PM and 10 PM, and 10 PM and 5 AM, respectively) [17]. Next, as a measure of new friendships, we calculated the number of accepted friend requests on Facebook. To operationalize social ties, we calculated the number of distinct people the patient messaged on Facebook and the total number of messages exchanged [34]. Finally, to measure the frequency of offline social interactions, we determined the number of posts that had location check-ins or cotagging with other people [31]. In general, we prioritized these specific items because they are well-validated and well-supported in the literature [31,33] in terms of revealing meaningful mental health insights from a clinical standpoint and in a clinical patient population.

#### Augmenting Existing Assessment Tools

We adopted 2 existing psychiatric assessment tools for the design of our prototype—the Hamilton Depression Rating Scale (HAM-D) [35] and the Global Functioning: Social (GF:S) [36]—because they are well-established tools that help clinicians track symptoms and the emotional, social, educational, and vocational functioning of patients. Typically administered in the form of interview-based assessments, there are 24 items in the HAM-D (eg, depressed mood and feelings of guilt) and 8 items in the GF:S (eg, Do you ever have problems or fallings out with friends?). For each item, clinicians ask questions to patients in person and observe their behaviors during the interview to assess their patients.

Although the assessment tools focus largely on offline behaviors—aspects that may not be covered in an individual’s Facebook activities—we designed a prototype that would enable clinicians to quickly assess social media–derived insights as an additional layer of collateral information on top of what might be accessible through the assessment tools. Our clinical collaborators felt that such complementary information gathered from patients’ social media activities can be useful. After deliberation and considering the social and emotional affordances of Facebook, we picked the 4 items from the HAM-D and 3 items from the GF:S (Table 1) that can be most reliably mapped to an analysis of patients’ Facebook data described earlier.

**Table 1:** Items, interview guides, and social media analysis in the prototype.

Items	Interview guide	Social media analysis
Depressed mood	How would you describe your mood in the past several days?	Number of depression-indicative posts
Suicidal thoughts	In the past several days, have you felt that life was not worth living, or that you would be better off dead?	Number of suicidal thought indicative posts
Insomnia	How have you been sleeping in the past several days?	Number of Facebook posts between midnight and 5 AM
Diurnal variation	In the past several days, have you noticed feeling worse at any particular time of day—such as in the morning or evening?	Number of depression-indicative posts by 4 time frames
New friends	Tell me about your social life. Do you have friends? If yes, how many friends would you say you have?	Number of accepted friend requests
Social ties	Are they casual or close friends?	Number of messages and recipients
Frequency of social interactions	How often do you see friends?	Number of posts with location and tagging

**Figure 1:**
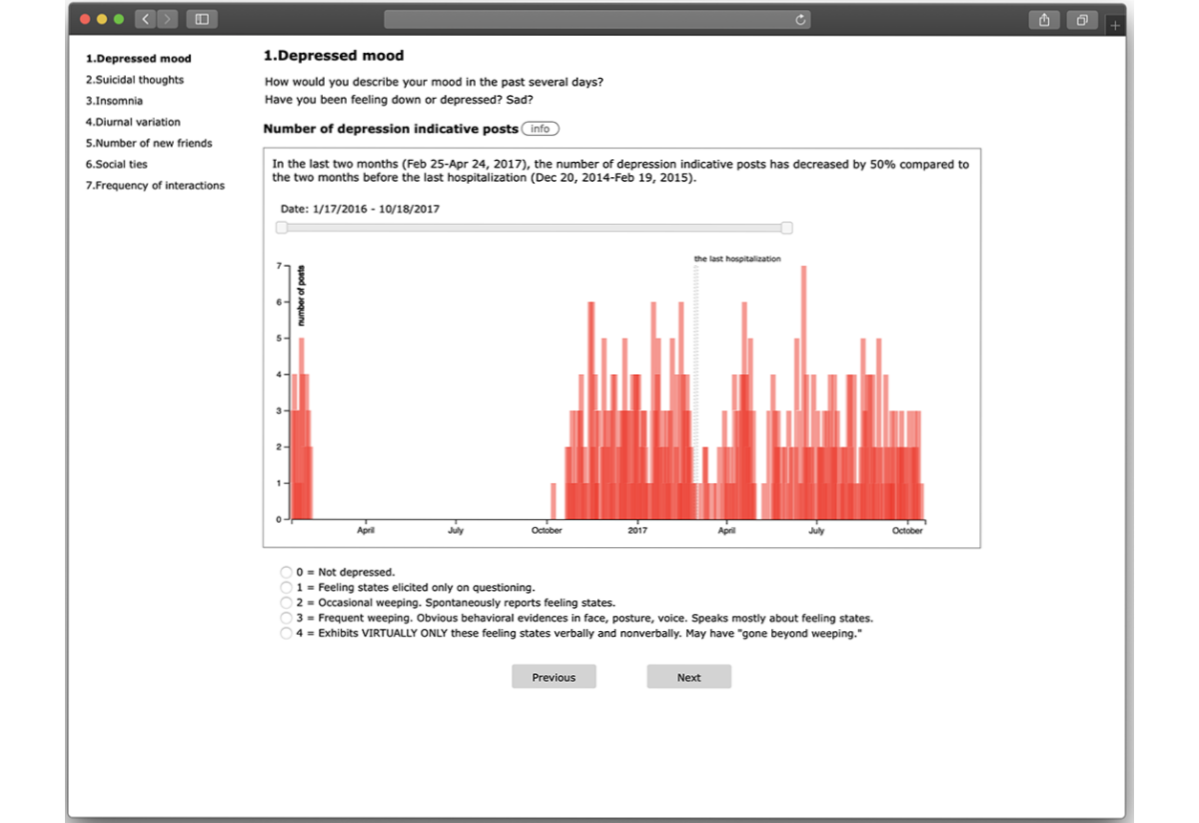
One view of the prototype.

We created an electronic version of a clinician-facing assessment dashboard that was augmented with social media analysis (Figure 1). We provided interview questions from the assessments on the top of the main page, so that clinicians can initiate the interview process. At the bottom of the screen, we placed anchored rating scales for the item, which come from either the HAM-D or the GF:S. Between the interview questions and the rating scales, the relevant social media analysis is displayed as collateral information. For example, for the depressed mood item, we added the number of depression-indicative posts from the patient’s Facebook data, visualized as a time series bar graph. The y-axis of this graph is the number of posts, and the x-axis of the graph represents time, that is, the time from account creation to the most recent activity. We added a range slider for the clinicians to adjust the time frame. Next to the title of the social media analysis, we added an information button that shows how we calculated the number of posts (eg, depression classifier for depression-indicative posts). Finally, we added a comparison between the last 2 months and 2 months before the last hospitalization of the same patient in plain text. This was to help clinicians find patterns that could indicate a change in symptoms. Additional screenshots of the prototype (eg, the suicidal ideation view) can be accessed in Multimedia Appendix 1.

### Qualitative Evaluation of the Prototype

#### Recruitment

To formatively evaluate the prototype powered by actual patients’ deidentified data, we used purposive sampling strategies to recruit mental health clinicians from a large, urban, behavioral health center located in the northeast of the United States. This research was approved by the institutional review boards of the relevant organizations.

To facilitate the recruitment process and to compensate for their participation, we provided a raffle for an iPad mini. We recruited 15 clinicians for individual interviews, and 13 of the 15 participated in a set of subsequent focus group sessions (3 sessions with 4-5 participants per session). In total, we had 8 psychiatrists, 5 clinical psychologists, and 2 licensed clinical social workers (Table 2). We grouped the focus group participants based on their availability. We recruited a heterogeneous group of clinicians because psychiatrists, psychologists, and social workers are highly collaborative in our study site.

**Table 2:** Participant demographics with their experience and gender. All focus group participants joined individual interviews before their focus group sessions.

Participants and title				Experience (years)		Gender
**Focus group 1**						
	P1	Psychiatrist (MD)	8		Female	
	P2	Clinical psychologist (PhD)	8		Female	
	P3	Licensed clinical social worker (MS)	6		Female	
	P4	Clinical psychologist (PhD)	7		Female	
	P5	Clinical psychologist (PhD)	11		Female	
**Focus group 2**						
	P6	Psychiatrist (fellow, MD)	4		Male	
	P7	Psychiatrist (fellow, MD)	6		Female	
	P8	Clinical psychologist (PhD)	20		Female	
	P9	Licensed clinical social worker (MS)	30		Female	
**Focus group 3**						
	P10	Psychiatrist (resident, MD)	1		Female	
	P11	Psychiatrist (resident, MD)	2		Female	
	P12	Psychiatrist (resident, MD)	2		Female	
	P13	Psychiatrist (resident, MD)	3		Female	
**Only interview**						
	P14	Psychiatrist (resident, MD)	3		Male	
	P15	Clinical psychologist (trainee, MS)	5		Female	

#### Procedure

First, to familiarize the participants with the prototype, we conducted an individual interview, where the participant explored the prototype and gave their feedback on it. Second, to envision future uses of the prototype, we conducted 3 focus group sessions where participants discussed the values and barriers of the prototype. These sessions were conducted between June and August 2019. The interview and focus group protocols are included in the Multimedia Appendix 2.

We interviewed participants in the same offices where they met their patients. Before obtaining informed consent, we provided an overview of the study. After the participants signed the informed consent form, they completed a demographic survey. We then asked questions about their work practices and their experiences with the patients’ social media. We demonstrated the prototype on a laptop. Next, the participants were asked to freely explore the prototype using a think-aloud protocol. We provided a vignette of a real patient, which was deidentified and edited for the study; the prototype displayed the same patient’s Facebook data. The clinician participants and the patients in the prototype were from the same behavioral health center; however, we did not check whether the participants had actually met the patients. Following the exploration, the participants answered follow-up questions regarding general feedback, compatibility with their work practices, and concerns and thoughts. The duration of the sessions ranged from 45 minutes to 80 minutes.

The subsequent focus group sessions were held for 55 minutes to 70 minutes. We explained the purpose of the focus groups and obtained informed consent from the participants. To refresh their memory of the prototype through a brief reintroduction, we asked about participants’ general feedback on the prototype. After that, the participants led the discussion comprising topics such as their willingness to use the prototype or their concerns.

#### Analysis of Qualitative Data

The sessions were audio-recorded with the participants’ permission, and the recordings were transcribed. The transcribed data were analyzed using thematic analysis [37]. The research team (DWY and BS) first deductively coded the data based on our research aims: (1) whether and for what purpose clinicians could incorporate the prototype into their work practices and (2) concerns and potential harms in its use and adoption in real-world clinical settings. We then inductively analyzed the data to identify particular patterns in the data. The initial codebook was iteratively reviewed during regular team meetings (DWY, MDC, MLB, and ARVM) until the team reached a consensus. The list of codes, their descriptions, and examples for each are included in the Multimedia Appendix 3.

## Results

### Overview

Participants considered the computational insights from patients’ social media to be helpful for clinician-patient collaboration. However, they also pointed out potential negative consequences and concerns that should be addressed in future technologies.

### Clinician-Patient Collaboration

While freely exploring the prototype, our participants voluntarily explained how they would like to use it to have a better conversation with their patients. In particular, they mentioned diverse collaborative situations where the prototype can be useful, such as when they explore directions for consultation and track changes in patients’ symptoms.

#### Collaborative Agenda-Setting

Our participants considered the prototype to be useful for them to collaboratively set an agenda with their patient—an approach strongly advocated in patient-centered and collaborative care models [38]. P11 suggested that reviewing the prototype with the patient at the beginning of the consultations could create awareness and concern about salient issues in a collaborative manner and the patient could feel that there is a more welcoming space where they can now “open up” and be “a contributor in [their] own treatment” (P5). The participants considered this a valuable outcome for enhancing their therapeutic alliance [39]:

If we have the patient in the office and were like, “Let’s spend 5 minutes and go through your data together.” And we look at the graphs together. And then point out, oh, it looks like at this period, you were posting a lot at nighttime, what was going on? And you just use it as a way to explore if something didn’t come up in the session. So obviously it could be used in a therapeutic way to enhance the therapeutic relationship.

P11

Participants pointed out that if they could have time to review the prototype before the consultations, it would help set the stage for what is to come during the session, and could affect the course, direction, and quality of care, including treatment decisions. In fact, such an approach could result in fewer *hidden* concerns at the end of the consultation (P9). For instance, clinicians could look for atypical or concerning patterns that might stand out when reviewing the prototype. If appropriate, during the session, they would then actively seek to know what happened during that time or why the patient posted something in particular on social media:

I could see myself using it this way: so if I’m meeting them on October 17 for an appointment, I’ll say, “Oh, have you had any suicidal thoughts in the last week?” And they say, “No.” And then, “I’m seeing on three occasions it looks like that on social media you were expressing something that maybe was concerning for suicidal thoughts, can you tell me about what these were if you remember them?” So I might use it to hone in on specific instances of suicidal thoughts.

P11

#### Communicating Symptoms

Communicating symptoms is one of the most important parts of clinician-patient collaboration [40]; however, it is often challenging because of the subjective nature of most mental illnesses and a lack of efficacious ways to monitor them longitudinally and in a fine granularity [41]. Therefore, our participants consistently pointed out that being able to gather more objective information regarding patients’ symptoms with the prototype could help both clinicians and patients to communicate symptoms:

Sometimes patients forget or don’t recall clearly for how long they had the symptoms, or how long they thought they have been secluding themselves in their room. Sometimes they don’t recall a rough timeline. But if you have the data in front of you in terms of how often have they been going out, and if you can clearly see a drop if they are a social person, but there has not been even a single tag, or they have not gone outside for a long while, you know you have to check into the situation.

P7

Participants further stated that sometimes patients may *minimize* certain symptoms, or as P11 noted, they can struggle to “recognize small changes,” in which case the prototype can help learn about the patients’ mental state. Difficulties in recognizing and communicating symptoms can be seen under certain conditions:

So maybe in a bipolar patient that would be more helpful. If they’re saying, “Oh yeah, I’m sleeping well” but they’re posting throughout the whole night, then you could see that their sleep patterns are off.

P12

Participants said that if the clinicians encountered such discrepancies, they would like to cautiously bring up the information from the social media analyses, as long as the patient is comfortable. In addition, they would explore the opportunity to address the gaps by having deeper conversations, such as by asking patients to unpack the foundations of this contradiction. P7 reported that patients “often live in denial” as and when they feel better intermittently. In that case, based on the prototype, it might be a meaningful psychotherapy probe to know “why the patient is not sharing what they have not shared,” that is, “was it just forgetfulness, or was it intentional on the part of the patient to hide certain things?” (P7).

However, participants also emphasized that the early moments of clinical interactions are important, as the tone of voice used by a clinician early in the visit is known to be indicative of satisfaction and compliance with treatment recommendations [38]. Therefore, they mentioned that there should be inconsistencies between what the patient says and what the prototype shows, they would approach this in a nonconfrontational manner, “do [so] subtly and bring it up to [the patients’] attention” (P3) and negotiate the appropriate time when this discussion may be timely.

As family members often engage in tracking symptoms and communicating with clinicians [42], our participants mentioned that the prototype may be useful in resolving conflicts between patients’ self-reports and collateral information from their family members:

Sometimes there is a tendency, by parents, when they do not recall clearly, of overgeneralizing things, like the patient has not talked to anybody in the last two or three months, has been really doing bad. But when you explore it clinically, the patient may tell you otherwise, even though the parent might deny it. But when you can see from their Facebook as well that they have been going out, they have been enjoying things that they had in the past, this can definitely correlate that fact.

P7

We considered the information from patients’ social media as another type of collateral information rather than a type of information that can replace any of the current information that clinicians may use. Our expectation is that clinicians will collectively consider every type of information available, including discreet conversations with patients. Our participants confirmed their interests in including information from social media in their decision-making process when appropriate.

### Incorporating the Tool in Current Work Practices

Different types of clinicians envisioned various ways to incorporate the prototype into their work practices. Clinical psychologists and licensed social workers (and some psychiatrists) mentioned that they would like to use the prototype similar to *homework assignments* [43]—cognitive behavioral therapy strategies suggest that homework assignments can help patients practice coping strategies and restructure dysfunctional beliefs. As our clinicians already discussed patients’ assignments at length during therapy sessions, they envisioned that the prototype could provide additional interesting discussion points. Although clinical psychologists and social workers preferred using the prototype to navigate their conversations, psychiatrists mentioned that they would like to check whether there were sudden changes after they modified some medication treatments. One of the psychiatrist participants, P14, mentioned that “being able to input when I started a medication would be very useful. And being able to even just note dose changes would be cool*.*”

Second, some participants suggested that reviewing the technology before consultations might be better from the perspective of patient-clinician engagement during consultations. This will ensure that conversations are not negatively disrupted with technology use, and it will prevent patients from “feeling neglected” (P10):

I think this is very valuable and I think there’s a very good role for that being incorporated in treatment. Personally, I might like having it here, something I review beforehand and then as needed or do a check in at a portion of the session where I’m like, “Let’s look together.” I just don’t know logistically if I’d want to keep [the prototype] in front of me the whole session. The patient might think, I don’t like how now my doctor is standing at a computer typing instead of talking to me.

P8

A third potential use of the prototype that some participants brought up included the possibility to learn about or keep track of specific patients’ symptom improvements or downturns when patients miss an appointment. P11 cited a case in which the prototype could provide timely feedback to the patient to enable them to self-reflect and be self-aware:

It’s often the case where patients don’t recognize small changes as much as maybe other people around them. So things like, they’re smiling more. They’re brighter, they’re more interactive, they’re talking for longer during the session. Those might be signs that their mood is improving. They might not notice it. So if there was some feedback I could give them, like “I notice that you’re looking a little bit brighter today or you’re a little bit better. And in fact, based on your social media use, it looks like you’ve been posting more positive things.“ That would be a great way to show it.

P11

P13, on the other hand, found that they could use the tool to connect with the patient in a timely fashion, even if an in-person consultation was not possible:

The irony of it is that when patients actually get sick is when they don’t come to see you. But if you are able to check in on them, like with this tool, that can be cool.

P13

### Potential Negative Consequences

#### Collaboration Versus Monitoring

Another conspicuous theme throughout the participants’ accounts concerns the potential negative consequences of the prototype. They pointed out that there will be a subtle line between collaboration and monitoring, and some patients might be negatively affected by the prototype. In addition, our participants expressed their negative opinions regarding the additional workload that the prototype may bring to clinicians.

Although participants voluntarily mentioned that the prototype can support collaboration and engagement during the consultations, some participants also expressed concerns; they provided scenarios where the patient may not choose to be an amicable party to the process. For instance, P9 mentioned that the mental illness experiences of certain patients may prevent their participation in the use of this prototype or there could be negative consequences:

The concern that everything that they do or even if they’re being monitored, big brother’s watching and even though consents are signed, I mean, paranoia is what, an irrational fear and they’re very vulnerable. So, it can go the other way too.

P9

Participants were concerned that the prototype’s abilities and clinical usefulness might be undermined by the Hawthorne effect [44], wherein patients may stop posting or begin to self-censor themselves on social media, knowing clinicians’ awareness of and access to this information:

It would be interesting how this will, this would modify their behavior on their social media considering the fact that they know now that, even their social media post has been given access to their clinicians. So like it’s being monitored. So like that might modify their behavior on the social media as well, either positively or negatively, depending on if they are seeking help or if they are seeking attention, in one way or the other. They might post more or they might start posting less.

P15

They further conjectured that patients who are less open and engaged during consultations would not consent to provide their social media data to the prototype, which is an important concern because openness and trust are critical to therapeutic alliance:

I feel like for the patients I’d want it, it’s those that I don’t trust and they’re not going to necessarily trust everyone with stuff on social media, and also kind of trust me to go look at their data, like to give me permission. So it would probably be looking at a lot of data from patients that we could just ask them the questions and they’ll be honest with us.

P4

#### Workload Issues

First, despite acknowledging it as a “technicality” (P7), participants were worried about the potential burden on their workloads. P11 felt that it may not always be feasible to review patients’ social media information before consultations in some programs, because in some clinical settings, patient loads are exceedingly high. In fact, the participants felt that reviewing additional data from the prototype might increase work and call for advanced training, either of which is likely to be impractical without adequate support from their institutions.

Participants also considered other areas of concern, such as general management of the prototype, explaining the scope of the prototype to (new) patients and its functioning to clinicians, maintaining informed consent from patients, getting help from the information technology staff to allow sustained use of the prototype, and ensuring that it is seamlessly integrated with other pieces of clinical information gathered by the institution—all of which they thought could lead to an increase in clinician burden. For instance, they pondered on who would educate the patients about this technology and manage issues, both technically related and patient-related. To this end, they thought they may be more willing to use this system when they are employees in a large hospital where someone else can handle the aspects surrounding the functioning of the system.

### Ethical Concerns

In addition to the potential negative consequences, our participants pointed out concerns that need to be addressed before this technology could be introduced in the clinical context.

#### Patient Privacy

When we introduced our prototype, we explained the privacy-related settings for it. The prototype’s data were collected with the patient’s consent for research purposes, and we envision that future technologies will actively seek patients’ consent to use their social media information in their treatment. Some participants mentioned that they were worried about patients’ privacy; however, they considered achieving patients’ consent to be the first step toward addressing such issues:

Also, the idea of someone being able to have their privacy of being able to poach these things without them having to have their doctors know about it all the time, but I guess if they’re agreeing to it, and that means they don’t mind.

P1

Our participants also provided keen insights regarding the sharing preferences of patients in a clinical context. P7 pointed out that even if the post is public, it is not clear whether the patient will be fine while sharing content with their clinicians. This idea opens up new questions about the difference between sharing a post with their friends and sharing a post with their clinicians. More importantly, this indicates that future consent procedures should be thorough in communicating the implications of sharing patients’ social media data with their clinicians. These ethical implications are explored in the Discussion section.

One of our measures to respect patients’ privacy, the design decision to not show the actual post in the visual representation, was well-received by clinicians. They pointed out that “not having a specific post is a little bit less invasive to the person’s privacy” (P8). However, it also raised a question about the trade-off between having the ability to review what the patient wrote and to protect patients’ privacy. This trade-off can be important, especially when they find a trend or pattern that might be relevant to the patient or their treatment. Multiple participants mentioned that they would like to read the post if the posts were flagged as suicidal ideation–indicative or depression-indicative, and they felt the pattern was important in the patient’s treatment. We envision that this tension regarding the granularity of shared data should be considered in future designs such as specific customization options for both clinicians and patients to decide the level of details that will be shared between them.

#### Credibility

There were 2 dominant credibility issues that participants thought could diminish trust in the prototype: if “Facebook posts and friend requests and everything correlate to actual life” (P12) and if an algorithm applied on top of these data can distinguish different contexts and intents behind specific posts. For instance, 1 participant repeatedly pointed out several times that they wanted to learn how the different algorithms worked and when they failed. Another participant mentioned a concrete scenario in which the algorithm may not be able to provide clinically meaningful insights because of the underlying gaps in psychometric validity:

And how does that algorithm delineate that certain posts are more likely to be related to depression versus others? Like if somebody had just posted they’re listening to some dark music, would it automatically pick that they’re suffering from low mood, that’s why they’re. Because sometimes people just write that on their Facebook post, they’re listening to this, and, like, that’s a part of dark music, or in general, sad songs.

P7

Another participant similarly mentioned that as the prototype does not distinguish between active and passive suicidal ideation, it cannot hint at the specific circumstances under which a patient may have shared a suicidal ideation–indicative post. A lack of this context may prevent clinicians from adjusting their treatment decisions based on the prototype, and it can be particularly difficult to ensure that the data augments patients’ and caregivers’ accounts, instead of eroding them:

Unless there’s behavioral action to back up what the person may have posted, I feel like it’s unclear how much conviction they had, and what they were saying, or whether it was just for attention. I think in terms of what is said, maybe more active stuff like, “I want to die; Life isn’t worth living anymore” will be more useful.

P10

Another participant further questioned relying on Facebook as the sole source of collateral information, as “people might [be] on a Facebook break” (P12). Ultimately, without subverting the utility of the prototype, participants said that, in the absence of an implicit level of trust or transparency in the functioning of the algorithms, they would consider the social media insights with caution:

I would probably trust the patient’s report more than I would trust the data from Facebook. I mean if the patient’s saying they’re doing totally fine and then they’re having a bunch of suicidal posts, I guess it’s a thing to bring up. But I wouldn’t necessarily feel that they’re suicidal because their posts say they are. I guess I would want more information, I wouldn’t just take it at face value. Because I know people post things for all different kinds of reasons. So I guess that’s my hesitancy, is this. . . and I got to trust what I see here.

P11

#### Liability

Liability issues may arise when clinicians have access to patients’ social media information via the prototype, which indicates an exacerbation of their symptoms, such as a crisis, but clinicians are not in a position to take any appropriate action:

There are posts which can be very critical. For example, “Oh, I’m going to kill myself now.” And then, if you don’t see this post, even though you have access to this information and you can access it at any time, are you responsible? So, there’s a lot more questions that come up if you have unlimited access to patient information at any time because the computer can then flag it. Right? That’s why I’m kind of my concerned more with like the legal and ethical stuff of how much you can/cannot be held responsible for.

P6

To this end, our participants wanted to clarify whether the Facebook data collection, and the analytics on top of it, happens in real time or if it is an episodic event that only happens when they meet their patients. On hearing that we intend the prototype to be used only when the clinician meets with the patient, participants thought the very act of volunteering their Facebook data may lead patients to think they are receiving 24/7 care; they may expect crisis mitigation resources all the time, outside of periodic clinician consultations. Participants felt that this could not only impact how clinicians currently manage crisis scenarios but also negatively impact their therapeutic relationship when patients’ concerns are not addressed as they occur. Consequently, participants highlighted the need for ethical and legal help from their institution:

I like to welcome that idea but I think if I’m in my private clinic or if I’m the only clinician then I would think about whether I would apply this, given various legal and ethical questions. Perhaps I would be more comfortable using it in the larger institution like here, in this hospital. Because they have legal rights and experts, and then if they say, “Okay, you can use it,” then, I’ll probably be more comfortable using it.

P6

Clinicians also brainstormed the liability around the aforementioned possible use case where they accessed patients’ social media information outside of consultations, such as when patients missed their consultations. We envision that, if the proposed technology is implemented in a real-world setting, it will be imperative to delineate when and in what circumstances (eg, during or outside of consultations) accessing these data is acceptable to the patients.

## Discussion

### Implications for Future Mental Health Technologies

Our work raises a vital question—how do we expect mental health treatment to be shaped in the future if a technology such as our prototype were to be used by clinicians?

#### The Future of Clinicians’ Work

Our findings reveal that our prototype can be a step forward in developing clinician-facing technologies that harness voluntarily shared patient social media data in mental health care delivery—a possibility advocated in prior work [33]. We found our prototype to be capable of providing a nuanced understanding of a patient’s unique illness course and clinical needs over time. Augmenting the short infrequent visits of today with our prototype, clinicians felt they could distill a stream of fluctuations in symptoms for a patient, calibrated against their baseline behaviors, and quantified against their past trends to detect subtle changes. Clinicians also appreciated the opportunity to correlate, corroborate, and contrast a patient’s clinical presentation with their behavior outside of the visit setting—a capability that can be particularly meaningful when a patient, because of cognitive impairment, has difficulty articulating their condition.

At the same time, clinicians expressed concerns regarding the credibility of the computational approaches that power the prototype. This was largely attributed to the fact that the acquisition of patient data was opaque to the clinicians and because providers thus far have not acquired patients’ social media data in the past. This is perhaps also unsurprising because many mental health clinicians are not used to using algorithm-generated information in their day-to-day work. Our participants repeatedly asked if social media reflected the patients’ actual mental health state, how the algorithms work, whether they were tested in a real treatment scenario, and when the algorithms failed. We noted that previous work has shown that people’s social media activities represent their actual selves [45], and we used previously validated social media measures of mental health in powering this tool [30,31,33]. However, we acknowledge that the prototype needs to persuade our potential users rather than relying on the support of previous research.

Technology has been reshaping the future of work in many domains [46,47]. Clinical work on mental health is no exception. These identified general needs for trustworthy algorithms are a core aspect of the future of clinicians’ work, which resonates with recent studies highlighting the importance of explainable, interpretable artificial intelligence and machine learning in health care [48,49]. Alongside these efforts, our findings also emphasize the need to consider structural changes in the future of clinicians’ work, specifically educating clinicians about the technology, not only to reduce the negative impact and potential harm attributed to poor credibility but also to make such technology more accessible to clinicians who may be conservative about new technologies.

We suggest the following calls to action accordingly:

1. Include resources to clarify the data collection process, when the system acquires the data, where the data are stored, and how the system accesses patient social media accounts, before both clinicians and patients experience this technology.
2. Provide the details of the algorithms both on-demand and contextualized in their demonstrated clinical efficacy, including evidence-based endorsement that can assure clinicians that the quality of the algorithms that power the technology is adequate.
3. Consider how technology education may be part of the psychiatry training paradigm, so that clinicians can gain some fluency in using a future version of this technology as an adjuvant tool in their clinical work.

#### The Patient-Clinician Therapeutic Relationship

We noted clinicians’ enthusiasm regarding how our prototype can help nurture early agenda-setting before in-person consultations. This feedback is particularly encouraging—in a busy clinical environment where time and throughput are paramount, clinicians may forego setting the stage at first based on patient feedback, to *get the work done* [50]. The clinicians in our study also thought that the information delivered through the prototype could be a helpful psychotherapeutic probe during consultations, wherein the patient’s clinical presentation on social media is reconciled with what they verbally report. Hence, we conjecture that the use of this tool can facilitate that the patient’s perceptions, needs, and concerns are considered appropriately by clinicians, in turn, helping to strengthen the therapeutic relationship between clinicians and patients.

However, our study also revealed potential scenarios in which the use of the tool may introduce new difficulties in managing the therapeutic alliance. According to the Agnew Relationship Measure [51], the therapeutic relationship between a patient and a clinician is defined by bond, partnership, confidence, and openness. During our study, we found that clinicians speculate on how the tool may negatively impact some of these core elements, such as patients’ openness to sharing sensitive information on social media, or their partnership in care, should this tool be introduced during consultations. Our clinician participants also felt unclear about what type of patient engagement was OK under various circumstances, and if patients felt comfortable with clinicians discussing with them highly sensitive information provided by the tool, such as that relating to suicidal thoughts. They also pondered the privacy and ethical challenges they might encounter when they find themselves obliged to connect with patients in case of a potential crisis scenario flagged by the tool but when the patient’s willingness to be contacted is unknown.

Even if these issues were to be mitigated in the future with deeper involvement of patients in exploring the use of the tool, a next step in this broader line of investigation, questions might arise about whether its use might undermine patients’ voice and autonomy, and their power in their treatment process. Although we emphasize that the role of the tool is not to replace patients’ self-reports but to augment them, it is not unusual for consented patients to feel that the tool would automatically replace the clinician’s judgments and decision-making. Patients may also feel insecure and think that their clinicians may disbelieve what they say, turning their conversations confrontational, especially when patients’ self-reports and social media data are not mutually consistent.

Here, we suggest the following calls to action to mitigate the challenges:

1. Consider provisions to continually negotiate patients’ involvement and agency in the use and functioning of this future technology throughout the treatment process.
2. Incorporate auxiliary risk management strategies to balance protecting patients’ privacy and clinicians’ obligation to reach out during moments of crisis revealed by the tool. These can include involving patient collateral or family members or liaising with additional safety resources (eg, patient groups and other health service providers).
3. Identify mutually negotiated terms between the clinician and the patient so that they agree when the technology is causing more harm than benefit or when benefit is no longer present.

#### Institutional Infrastructures

Finally, our findings indicate the need to consider creating adequate institutional support to co-ordinate a sustainable ecosystem of stakeholders in both the deployment and maintenance of the prototype. The goal will be to assuage concerns that our study raised regarding compatibility with their existing workflows, increased burden on the clinicians, and liability and perceived lack of resources to support the use of the technology. For instance, our clinician participants noted the moral and professional quandary when they discovered alarming patterns, such as active suicidal ideation through this technology. Relatedly, they felt that the timing of when, during an ongoing consultation, to bring up the social media analyses is critical, but currently there is little counsel on it. Others expressed reservations, wondering if using the tool during an ongoing consultation was a good idea at all because it can potentially be distracting and rude, and take away the much-needed focus and eye contact desired during a conversation with a patient. Therefore, the technology will also have to be appraised continually through institutionally enforced policies so that trust and confidence in its use are maintained.

We offer the following calls to action in light of these observations:

1. Facilitate collaboration of diverse institutional stakeholders, such as management and information technology personnel, legal staff, and clinicians and patient advocacy groups, to develop institutional policies surrounding the technology.
2. Develop institutional provisions that advise clinicians on how to attend to any potential crisis discovered by the tool and standardize professional guidelines within the institution around what type of use of the technology in the context of a patient’s care is acceptable.
3. Frame overarching policies governing what are the goals of care improvement when this technology is used and how efficacy and safety can be assessed throughout the period of a patient’s care.
4. Suggest medical institutions to consider creating a new role to enable better assimilation of such a technology in mental health care—a “technology coach for mental health” or a “digital navigator” [52,53], similar to the notion of a patient navigator in cancer care [54] or a technology coach in web-based education [55], who can serve as an interface between the technology and the clinician, and the technology and the patient.

### Ethical Implications

Although the tool we discuss in this study exclusively focuses on scenarios where a patient would have consented to have their data collected and used in the prototype for clinicians’ use, we see remaining ethical concerns around the concept of using social media at the point of care.

First, we acknowledge that managing consent is a murky topic. Informed consent has been widely accepted as a legal and ethical requirement for most health care transactions; however, researchers have been reflecting on informed consent practices, especially on how much the participants should understand, how explicit their consent should be, and the delicate consideration of a patient’s authenticity of choice (ie, voluntarism) [56], for instance, when a patient feels potential coercive pressures to incorporate this technology into their care, or social pressures to engage with new technology. Furthermore, patients may not fully appreciate what they are revealing when they consent, so they may share social media activities that they would otherwise choose not to share with their clinicians. The patient may also misunderstand that there might be a disadvantage if they do not participate in the sharing program. To address these problems, we need to consider a *sustained informed consent* [57] procedure in which someone will continually revisit informed consent with the patients, providing detailed information about both the sharing process and the voluntary nature of the program, as well as potential clinical and ethical harms.

Finally, we should consider the legal perspectives of future technologies. According to the Food and Drug Administration Safety and Innovation Act [58], most clinical decision support that delivers knowledge, person-specific information, and intelligently filtered information to clinicians and patients is not regulated by authorities. In addition, the source of data that will power this technology—social media—is not considered protected health information. However, because computer-aided detection or diagnosis can be considered a medical device, it raises an important question about whether such future technology should be overseen. Technology regulations also need to be considered by researchers and technology designers. We argue that even if the technology is not considered a medical device, the Food and Drug Administration Safety and Innovation Act’s recommendations should be considered.

### Limitations and Future Work

We note that our work suffers from some limitations, which constitute avenues for future research. First, we recruited patients from one health center and included a limited number of clinicians; therefore, the results may not be generalizable. Second, our participants explored the prototype without real interactions with their patients. By deploying the technology during actual appointments, future research can assess its ecological validity.

Finally, our study did not explore patients’ opinions on the technology we proposed, although our study and the design decisions behind the prototype were situated in positive attitudes expressed by patients in sharing their social media data for diagnostic and treatment purposes [59,60]. We note here that this study is the first of a series of studies that plan to understand the potential and barriers of social media–powered technologies to support mental health treatment. We plan to explore this from a multistakeholder perspective, an important one being the patients. We believe that clinicians’ feedback is a natural first step in this line of investigation. Using social media for mental health without the clinicians’ guidance or support can be dangerous [24], and a lack of demonstrated clinical utility and buy-in from clinicians is likely to render subsequent studies less meaningful [61]. As argued by Baier [23], the potential harm of inappropriate social media use could be seen as a violation of this principle from indirectly encouraging boundary crossings to burdening patients with unnecessary information that could compromise the therapeutic environment. This motivated us to consider interviewing clinicians first in the work presented in this study. As a next step, our goal is to explore patients’ attitudes toward this potential technology.

### Conclusions

This study presents a qualitative design study, including the design and evaluation of a prototype, to explore mental health clinicians’ perspectives regarding a future technology that delivers computational insights derived from consented patients’ social media. Our findings reveal the promise of the prototype beyond its compatibility with work practices. At the same time, the participants reported concerns and potential barriers to the new technology. The design of such technology should address the potential negative consequences and ethical concerns regarding credibility, liability, and institutional support. Our findings necessitate future research exploring patient perspectives on using computational insights from their social media in the context of their treatment.

## Appendix

Multimedia Appendix 1 Screenshots of the prototype.

Multimedia Appendix 2 The interview and focus group protocols.

Multimedia Appendix 3 The list of codes, their description, and examples.

## Abbreviations

GF:S: Global Functioning: Social
HAM-D: Hamilton Depression Rating Scale
