# Supplementary material for: Clinician Perspectives on Using Computational Mental Health Insights From Patients’ Social Media Activities: Design and Qualitative Evaluation of a Prototype
Source: JMIR Ment Health. 2021 Nov 16;8(11):e25455. doi: 10.2196/25455 (PMC8663497; doi:10.2196/25455)
Supplement: Multimedia Appendix 1 [file mental_v8i11e25455_app1.pdf]

Supplementary Material: Screenshots of Prototype

1.Depressed mood
2.Suicidal thoughts
3.Insomnia
4.Diurnal variation
5.Number of new friends
6.Social ties
7.Frequency of interactions

1.Depressed mood

How would you describe your mood in the past several days?
Have you been feeling down or depressed? Sad?

Number of depression indicative posts info

In the last two months (Feb 25-Apr 24, 2017), the number of depression indicative posts has decreased by 50% compared to the two months before the last hospitalization (Dec 20, 2014-Feb 19, 2015).

Date: 1/17/2016 - 10/18/2017

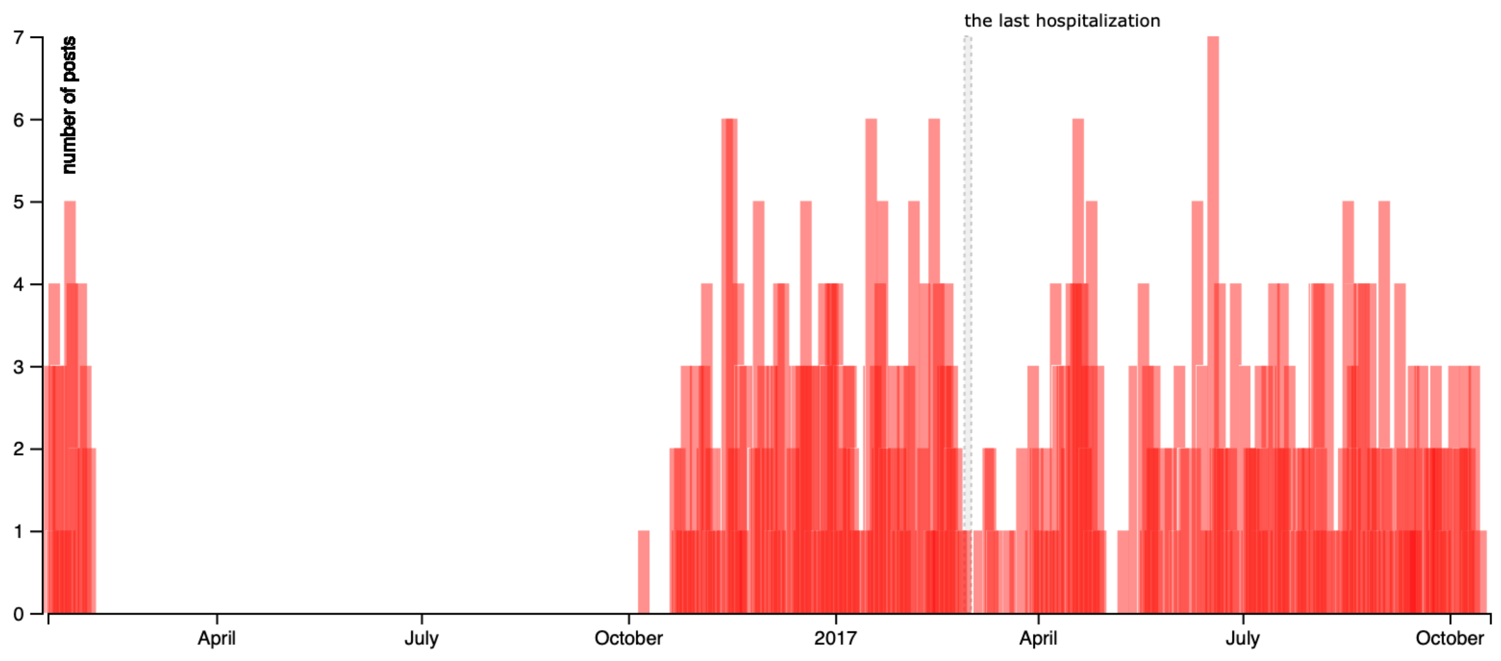

- 0 = Not depressed.
- 1 = Feeling states elicited only on questioning.
- 2 = Occasional weeping. Spontaneously reports feeling states.
- 3 = Frequent weeping. Obvious behavioral evidences in face, posture, voice. Speaks mostly about feeling states.
- 4 = Exhibits VIRTUALLY ONLY these feeling states verbally and nonverbally. May have "gone beyond weeping."

Previous   Next

1.Depressed mood
2.Suicidal thoughts
3.Insomnia
4.Diurnal variation
5.Number of new friends
6.Social ties
7.Frequency of interactions

2.Suicidal thoughts

In the past several days, have you felt that life was not worth living, or that you'd be better off dead? What about thoughts of hurting or killing yourself?

Number of sucidal thought indicative posts info

In the last two months (Feb 25-Apr 24, 2017), the number of posts expressing suicidial thoughts has not changed compared to the two months before the last hospitalization (Dec 20, 2014-Feb 19, 2015).

Date: 1/17/2016 - 10/19/2017

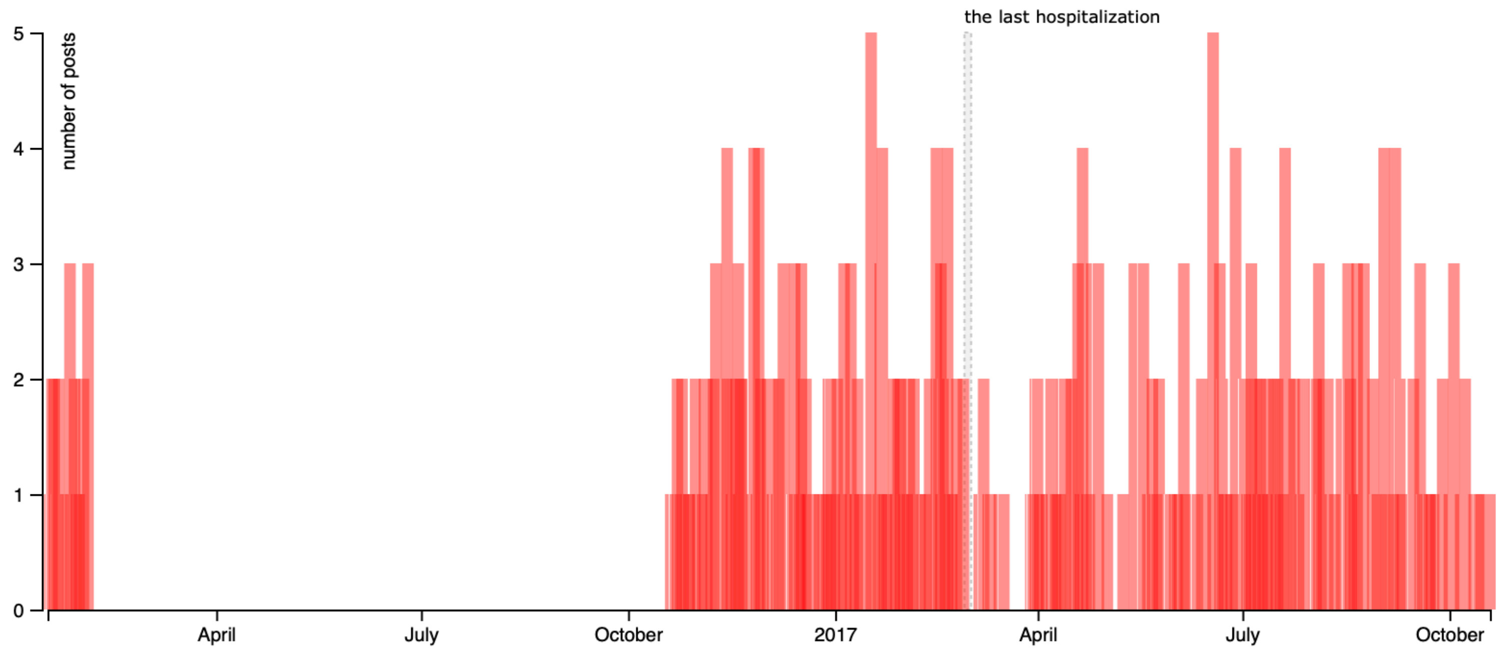

- 0 = Absent.
- 1 = Feels life is empty or not worth living.
- 2 = Recurrent thoughts or wishes about death of self.
- 3 = Active suicidal thoughts, threats, gestures.
- 4 = Serious suicide attempt.

Previous   Next

1.Depressed mood
2.Suicidal thoughts
3.Insomnia
4.Diurnal variation
5.Number of new friends
6.Social ties
7.Frequency of interactions

3.Insomnia

How have you been sleeping in the past several days?

Number of Facebook posts between 12am to 5am

In the last two months (Feb 25-Apr 24, 2017), the number of posts between 12 am to 5am has decreased by 100% compared to the two months before the last hospitalization (Dec 20, 2014-Feb 19, 2015).

Date: 1/17/2016 - 10/19/2017

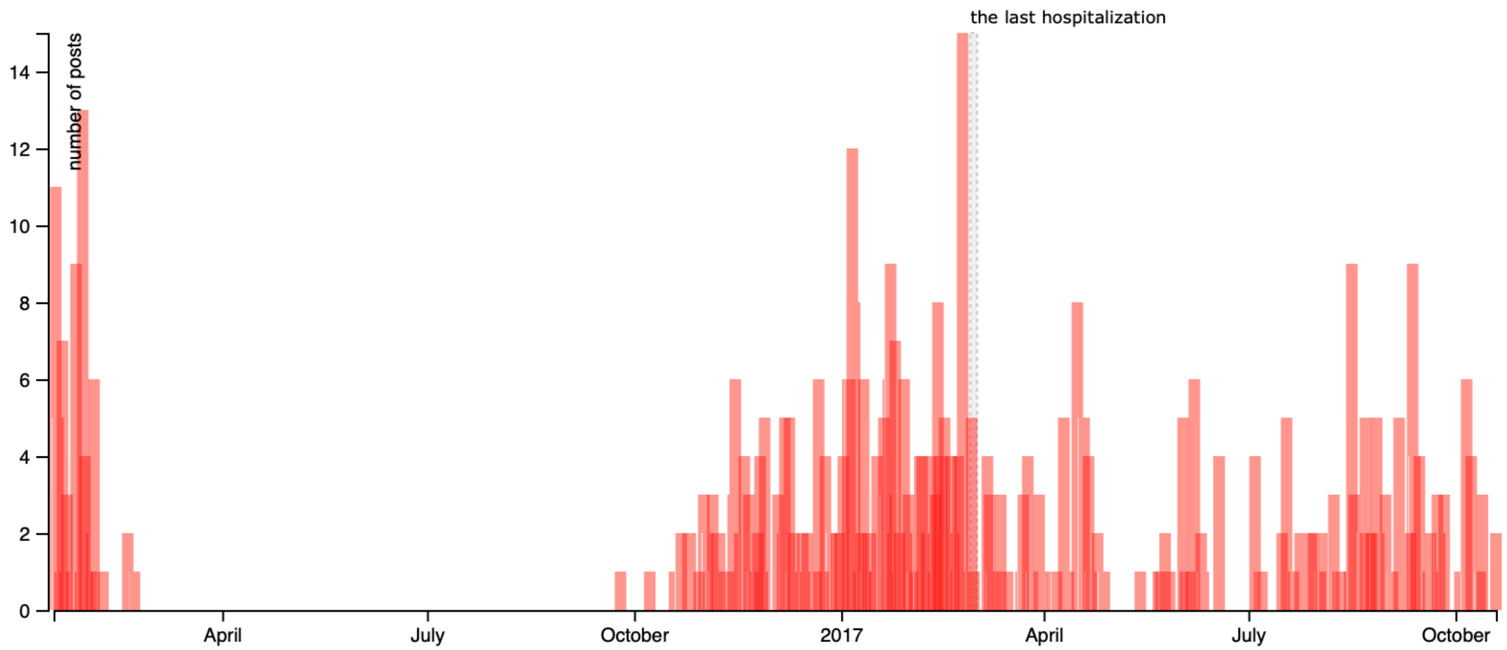

- 0 = Absent.
- 1 = Mild, infrequent; more than 1/2 hour occasionally.
- 2 = Obvious and severe; more than 1/2 hour usually.

Previous Next

1.Depressed mood
2.Suicidal thoughts
3.Insomnia
4.Diurnal variation
5.Number of new friends
6.Social ties
7.Frequency of interactions

4.Diurnal variation

In the past several days, have you noticed feeling worse at any particular time of day - such as in the morning or evening?

Number of depression indicative posts by 4 time frames info

In the last two months (Feb 25-Apr 24, 2017), the number of depression indicative posts has/has not changed in the following manner when compared to the two months before the last hospitalization (Dec 20, 2014-Feb 19, 2015):
Morning (5am-12pm): has decreased by 92%
Midday (12pm-5pm): has decreased by 100%
Evening (5pm-10pm): has not changed
Night (10pm-5am): has not changed

Date: 1/17/2016 - 10/19/2017

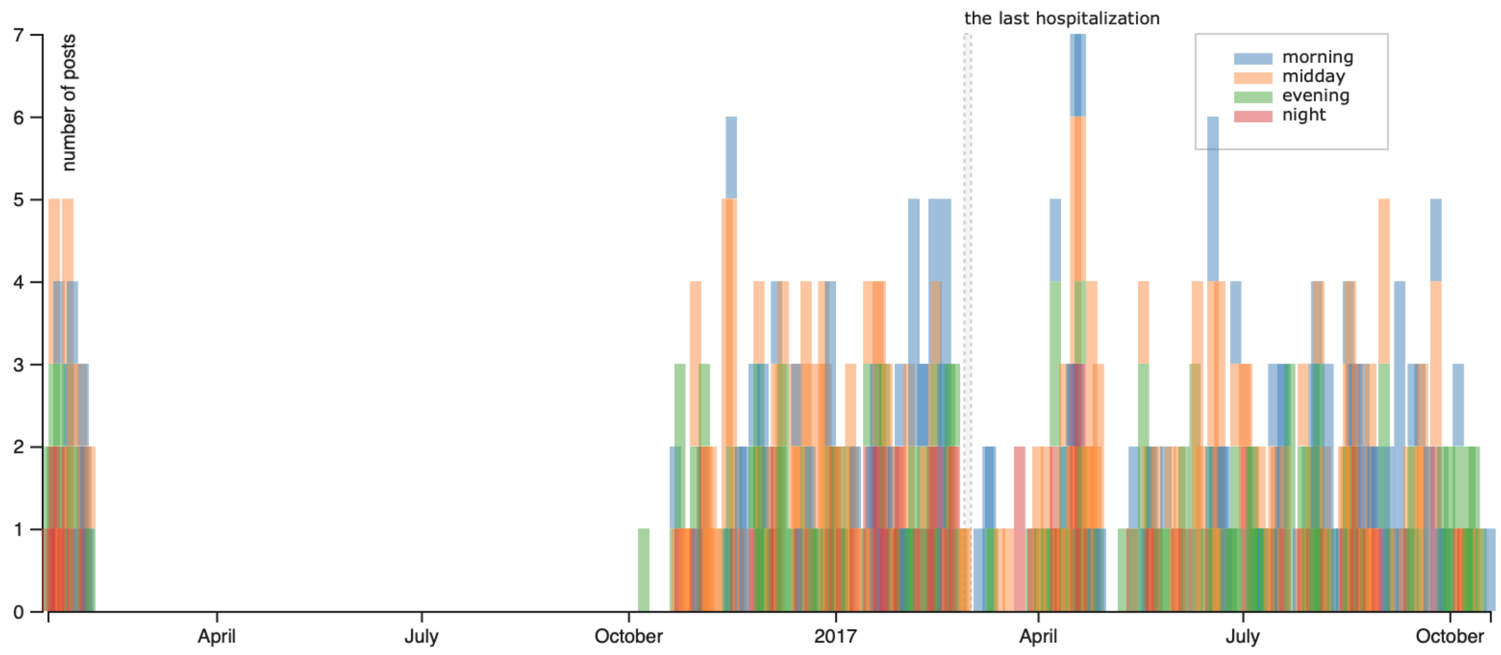

- 0 = Absent.
- 1 = Mild.
- 2 = Severe.

Previous Next

1.Depressed mood
2.Suicidal thoughts
3.Insomnia
4.Diurnal variation
5.Number of new friends
6.Social ties
7.Frequency of interactions

5.Number of new friends

Tell me about your social life. Do you have friends? If yes, how many friends would you say you have?

Number of accepted friend requests (info)

In the last two months (Feb 25-Apr 24, 2017), the number of newly added Facebook friends has decreasd by 100% compared to the two months before the last hospitalization (Dec 20, 2014-Feb 19, 2015).

Date: 1/17/2016 - 10/19/2017

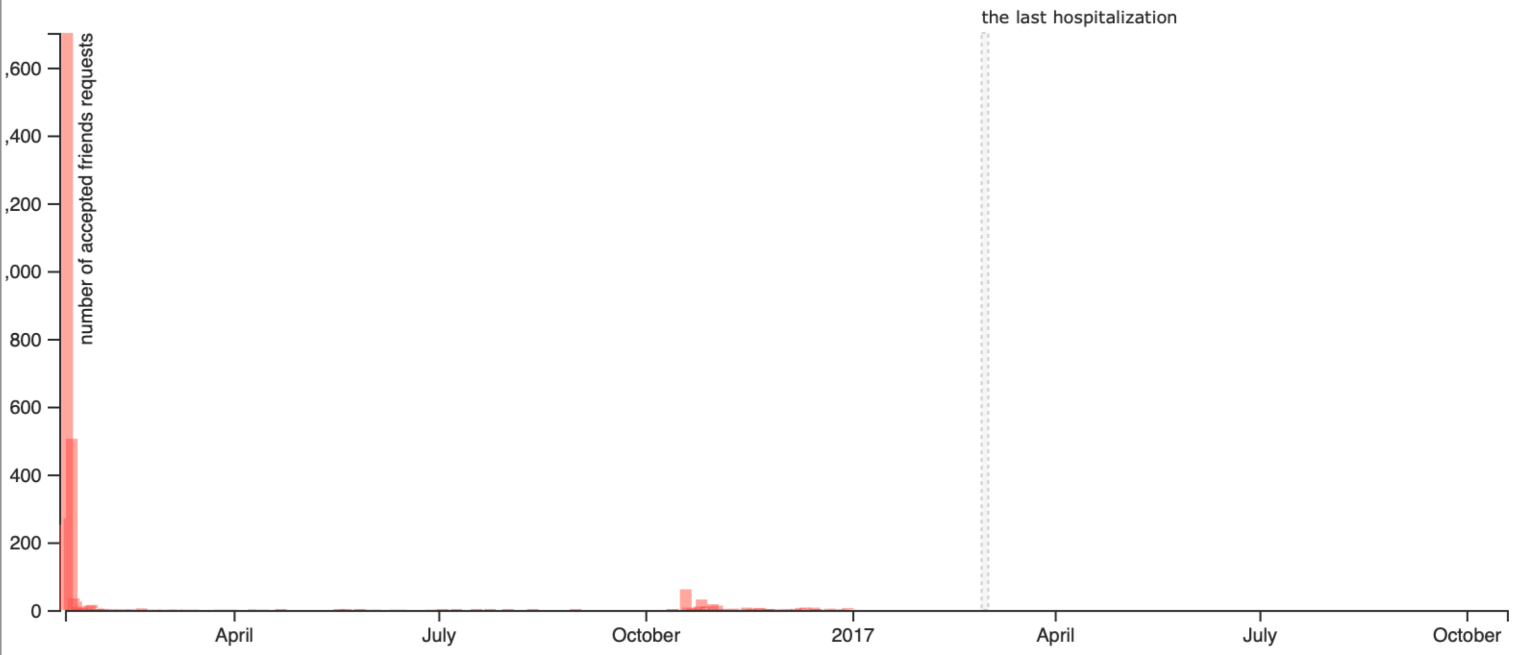

- 1 = Extreme social isolation.
- 2 = Unable to function socially or to maintain any interpersonal relationships.
- 3 = Marginal ability to function socially or maintain interpersonal relationships.
- 4 = Major impairment in social functioning.
- 5 = Serious impairment in social functioning.
- 6 = Moderate impairment in social functioning.
- 7 = Some persistent mild difficulty in social functioning.
- 8 = Some transient mild impairment in social functioning.
- 9 = Good functioning in all social areas, and interpersonally effective.
- 10 = Superior functioning in a wide range of social and interpersonal activities.

Previous Next

1.Depressed mood
2.Suicidal thoughts
3.Insomnia
4.Diurnal variation
5.Number of new friends
6.Social ties
7.Frequency of interactions

6.Social ties

Are they casual or close friends? If only casual — are they school/work/on-line friends only? Are they more like acquaintances?

Number of messages and recipients (info)

In the last two months (Feb 25-Apr 24, 2017), the total number of messages has decreased by 69% compared to the two months before the last hospitalization (Dec 20, 2014-Feb 19, 2015).
In the last two months (Feb 25-Apr 24, 2017), the total number of recipients has decreased by 50% compared to the two months before the last hospitalization (Dec 20, 2014-Feb 19, 2015).

Date: 1/17/2016 - 10/19/2017

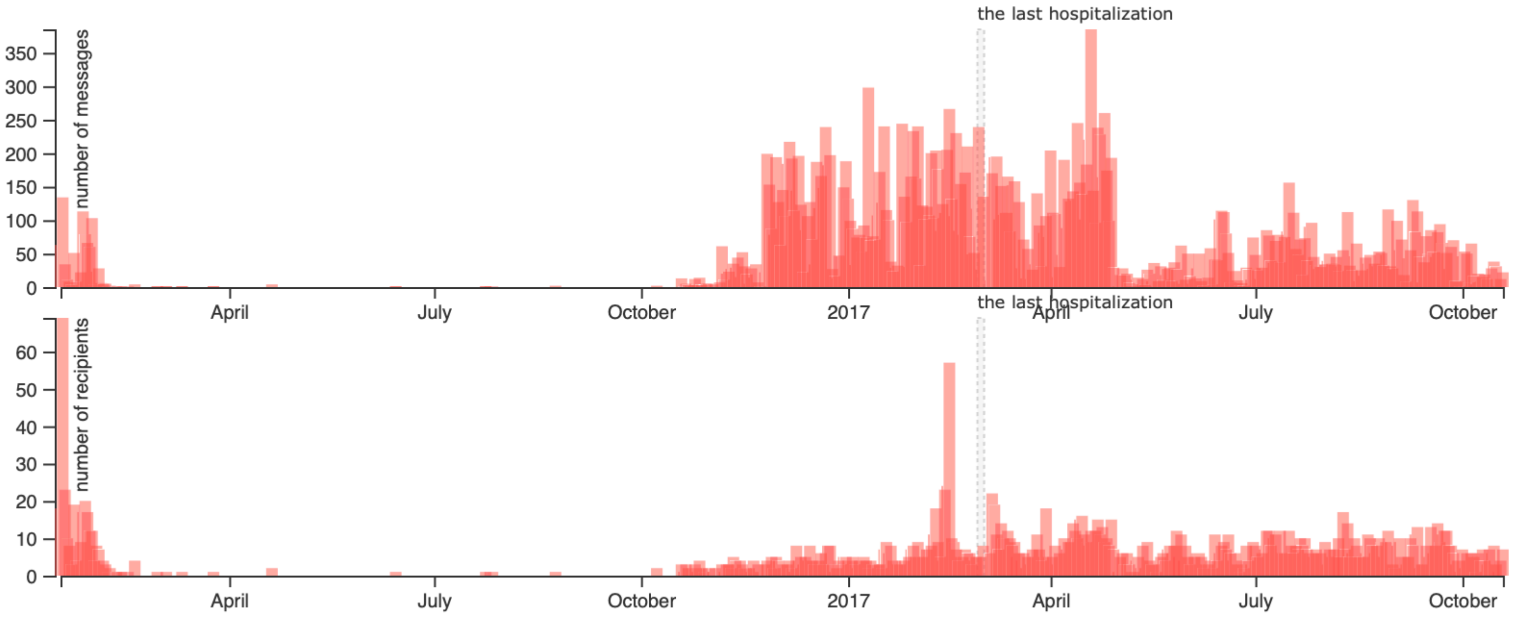

1 = Extreme social isolation.
2 = Unable to function socially or to maintain any interpersonal relationships.
3 = Marginal ability to function socially or maintain interpersonal relationships.
4 = Major impairment in social functioning.
5 = Serious impairment in social functioning.
6 = Moderate impairment in social functioning.
7 = Some persistent mild difficulty in social functioning.
8 = Some transient mild impairment in social functioning.
9 = Good functioning in all social areas, and interpersonally effective.
10 = Superior functioning in a wide range of social and interpersonal activities.

Previous   Next

1.Depressed mood
2.Suicidal thoughts
3.Insomnia
4.Diurnal variation
5.Number of new friends
6.Social ties
7.Frequency of interactions

7.Frequency of intereactions

How often do you see friends? Do you see them outside of work/school?

Number of posts with location and tagging info

In the last two months (Feb 25-Apr 24, 2017), the total number of posts with location and tagging have decreased by 63% compared to the two months before the last hospitalization (Dec 20, 2014-Feb 19, 2015).

Date: 1/17/2016 - 10/19/2017

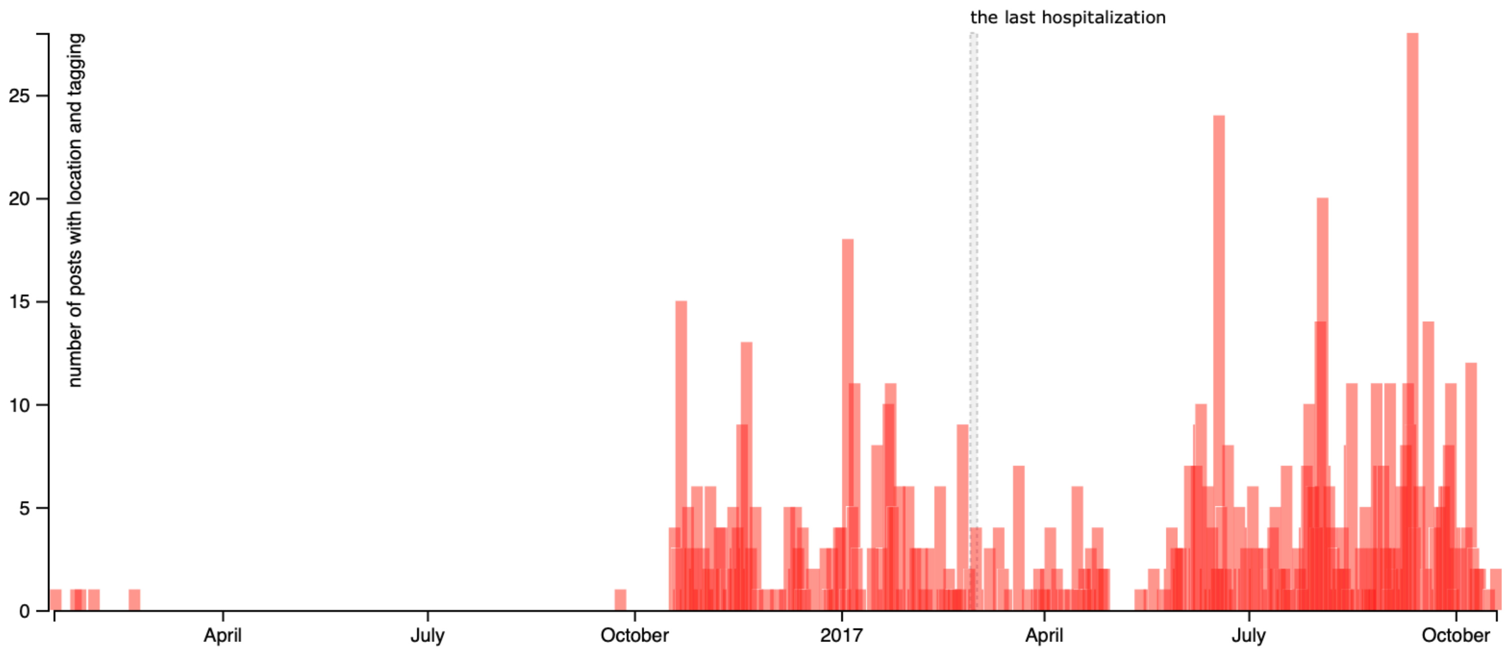

- 1 = Extreme social isolation.
- 2 = Unable to function socially or to maintain any interpersonal relationships.
- 3 = Marginal ability to function socially or maintain interpersonal relationships.
- 4 = Major impairment in social functioning.
- 5 = Serious impairment in social functioning.
- 6 = Moderate impairment in social functioning.
- 7 = Some persistent mild difficulty in social functioning.
- 8 = Some transient mild impairment in social functioning.
- 9 = Good functioning in all social areas, and interpersonally effective.
- 10 = Superior functioning in a wide range of social and interpersonal activities.

Previous Next
