# Supplementary material for: Clinician Perspectives on Using Computational Mental Health Insights From Patients’ Social Media Activities: Design and Qualitative Evaluation of a Prototype
Source: JMIR Ment Health. 2021 Nov 16;8(11):e25455. doi: 10.2196/25455 (PMC8663497; doi:10.2196/25455)
Supplement: Multimedia Appendix 2 [file mental_v8i11e25455_app2.pdf]

Individual Interview with Prototype

Steps	Apparatus	Time (total 60 min)	Contents/questions
Introduction		10 min	● Why social media?<br>● The purpose of this project/session<br>● Overview of this individual interview
Informed consent	consent document	5 min	● An interviewer will go through the consent document and answer any questions that may arise
Demographic survey	survey (paper)	5 min	● Specialty, career level, technology usage, prior experience with patients' social media
Exploratory free use of the prototype	prototype<br>think aloud	15 min	● An interviewer will demonstrate how to use the tool<br>● After that, they are asked to freely explore the tool with a patient vignette using think aloud protocol
Exit interview	Semi structured interview questions	25 min	● Questions about work practice<br>● Questions about potential use of the prototype<br>● Questions about concerns

Introduction
Thank you for electing to participate in this study. My name is XX and I'll be facilitating this session today.

Foremost, I'd like to briefly explain this research project. Ultimately, we aim to design a new technology that can help mental health clinicians and patients at the point of care. Specifically, we are interested in utilizing insights we can draw from patients' social media data.

Previous research in computer science has proven that social media data, such as what is written on Facebook, can reveal some valuable aspects of mental attributes, such as affect or depression. Analyses on social media data can be powerful in understanding one's mental health status in many ways as it has a lengthy history and is less subject to recall bias. Therefore, we expect that the inferred mental health status based on linguistic analyses of social media usage can be useful collateral information which can help mental health clinicians to have a better picture of their patients. For example, from the linguistic analyses of social media, we can learn whether this person is vulnerable to mental health conditions, such as depression, post-traumatic stress disorder, and schizophrenia. To do that, we analyze the social media posts to understand mood and emotional states (e.g., fear, anxiety, joviality), cognition (counting the number of words that reveal their cognition), and language patterns, such as function word use or interpersonal pronoun use. We can also learn their behavioral patterns from social media posts, such as how they interact with others on and offline using social media, the quantity and quality of the private messages they exchange, and their circadian rhythms.

However, as the insights from social media have not been introduced in a clinical setting, we need to learn whether social media analyses can be valuable to clinicians and how we can develop a usable interface of social media analyses for clinicians. To learn those, we would like to hear mental health clinicians' opinions regarding potential usefulness, potential barriers, and areas of improvement for a new technology we would like to introduce.
Today, if you consent to participate, we will do a short demographic questionnaire, followed by an introduction to the prototype, which is basically a computerized HAM-D and Global Functioning assessment utilizing real patient social media analyses. I will walk you through the prototype and ask you to freely explore it for about 15 minutes. Finally, you will be asked to answer some follow-up questions from me.

Please note that the purpose of this session is not to test you; we would like to test our prototype. If you cannot understand what the prototype provides, it means we need to improve the prototype to be easier to comprehend.

Informed Consent Procedure
We will conduct this session with your consent. For that purpose, we've prepared this consent form for you to look over. Please take as much time as you need to read this document. You can ask questions at any time. If you agree to participate in this project, please sign at the bottom of the document.

Before you sign the document, I want to highlight this part, "Use of Photographs, Audio, or Video Recording." As described here, we would like to audio record this session because we will conduct a systematic qualitative analysis on the transcript of the recordings. We will do our best to protect your privacy during collection and analysis. For example, this audio recorder is a password protected, encrypted one. After being transcribed, the recordings will be deleted. We will delete the identifiable parts (i.e., names) from the transcripts and use alphanumeric codes to refer to participants.

As soon as you sign the form, I'll begin audio-recording. If you choose not to be audio-recorded, I would like to thank you for your time, but I'm afraid that we cannot move forward (end of session.)

Demographic Survey
Thank you for signing the consent form. I'd like to ask you to fill out this demographic survey. It will be used in an aggregated form in our future research paper and any personal information will not be shared outside of the research team.

Walkthrough of Prototype

Thank you for sharing your information. Now I'd like to walk you through our prototype. This is a website that you can access using your machine and a web browser. Let's assume that you are accessing this web page using your machine.

The purpose of this tool is to deliver mental health insights derived from patients' social media data, in this case, Facebook posts. If we analyze language patterns of what patients have written on social media, we may be able to discern interesting information, such as whether they have expressed a depressed mood or not. We believe this information can augment current methods, such as interviews and questionnaires, and can function similarly to collateral information.

So this tool is to aid your interview process. We picked 7 categories from either HAM-D or GFSS that can be well supported through the patients' social media use patterns. The top part is an interview guide we borrowed from HAM-D and the bottom part is an anchored rating scale for that item. Between these, in the box, this is the social media analysis as collateral information.

Let me explain the social media analysis in this prototype. Firstly, we used the Facebook data of a real patient. We obtained their consent when we acquired this data and we envision that we will only use the social media data of those who have provided informed consent for this process. And we conducted a simple data analysis on the data, such as the number of depression indicative posts.

So we suggest that you may be able to use this tool when you interview your patients and you can look at some insights from their Facebook data to see if this information contradicts or supports what is being said during sessions. It can then be used as a further avenue of discussion at the point of care. But this is just one of the possible uses of the social media insights, and we believe there are other ways to use this, specifically ways to better suit your workflow. I'll ask for other possible uses of the information after you explore this prototype.

We have prepared a vignette of the patient who shared this social media data. The vignette is anonymized and edited to protect personal health information but it reflects real patient medical history, so it can help you to understand the prototype.

Do you have any questions so far? If not, I'll let you explore this tool for about ten to fifteen minutes.

Exploratory Free Use of Prototype (Think-aloud protocol)

Now, I would like to ask you to delve into this tool. Please feel free to look at and click anything you're interested in. You can also ask questions if you would like to do so. Please verbalize your thoughts as you work with the prototype. I've set aside 15 minutes for this, but please let me

know if you are ready to move onto the next step before then, or if you would like to have more time to look it over.

Exit Interview

Do you have any comments you would like to make about the prototype? Is there a portion of the questionnaire itself you would like to expand on in greater detail?

Additional questions (if not answered):

- General experience
  - What do you think about this prototype?
- Ease of use
  - Were you able to discover how to read and use the system?
  - Were there any areas in which you struggled with the prototype? Were there any aspects of the prototype you would choose not to use, if it were available to you today?
  - Could you explain what was the most difficult part to understand in the prototype?
  - Did you refer to the social media analysis when you rated the item?
- Work practice
  - Could you tell me about your typical day as a clinician?
  - Given your daily work practices, what do you think about the prototype?
  - What kinds of collateral information do you usually use? How do you obtain them? How do you use them in your practice?
- Burdens/barriers
  - Do you see any barriers which may prevent you from using this prototype in your everyday practice?
    - job constraints - do you think this prototype could fit into your work practice?
    - time constraints - how much time do you think you can invest in using this prototype in your everyday activities
  - Do you see any possibilities where patients may feel uncomfortable using this prototype in their treatment?
    - Do you think the data collection process is transparent enough to make patients comfortable?
    - Do you think the prototype is transparent enough to make patients comfortable?
- Concerns and thoughts
  - Do you have any concerns/thoughts regarding using social media analyses in your current work practices?
  - Do you have any ideas which may assuage some of your concerns?

Focus Groups Guide

Steps	Apparatus	Time (total 60 min)	Contents/questions
Introduction	prototype	5 min	● A demo as a reminder of our prototype
Informed consent	consent document	5 min	● A facilitator will go through the consent document and answer any questions that may arise
Free discussion	semi-structured question guide	50 min	● A facilitator will lead the discussion using the question guide:<br>○ Participants will interact with each other to advance their discussion<br>● When they want to review the prototype again, they can interact with the prototype using the laptops in the room.

Welcome
Thank you for electing to participate in this study. My name is [facilitator name]. You have been asked to participate as your point of view is important. I realize you are busy and I appreciate you taking the time to meet with me again.

Introduction
This focus group discussion is designed to learn your opinions regarding a prototype which will provide insights from patient social media as a form of collateral information when you are interacting with your patients. I would like to demo the prototype first.

Informed Consent Procedure
We will conduct this session with your consent. For that purpose, we've prepared this consent form for you to look over. Please take as much time as you need to read this document. You can ask questions at any time. If you agree to participate in this project, please sign at the bottom of the document.

Before you sign the document, I want to highlight this part, "Use of Photographs, Audio, or Video Recording." As described here, we would like to audio record this session because we will conduct a systematic qualitative analysis on the transcript of the recordings. We will do our best to protect your privacy during collection and analysis. For example, this audio recorder is a password protected, encrypted one. After being transcribed, the recordings will be deleted. We will delete the identifiable parts (i.e., names) from the transcripts and use alphanumeric codes to refer to participants.

As soon as you sign the form, I'll begin audio-recording. If you choose not to be audio-recorded, I would like to thank you for your time, but I'm afraid that we cannot move forward (end of session.)

Anonymity

Despite being audio-recorded, I would like to assure you that the discussion will be anonymous. The audio recordings will be safely kept in a password-protected, encrypted device until they are transcribed word for word, then they will be destroyed. The transcribed notes of the focus group will contain no information that would allow individual subjects to be linked to specific statements. I, and the other focus group participants would appreciate it if you would refrain from discussing the comments of other group members outside the focus group. If there are any questions or discussions that you do not wish to answer or participate in, you do not have to do so; however please try to answer and be as involved as possible.

Questions:

- Ideas regarding new technologies
  - What are the barriers you have when you interact with your patients?
  - What are the barriers you feel when you interact with other stakeholders? (co-workers, patients' family members, etc)
  - Do you have any problems/concerns when you use technologies at your workplace?
  - If you can access patient social media data/analyses similar to this prototype, what kinds of visualizations/systems/technology can help you to better work with your patients?
  - If you were allowed to suggest any kinds of technology which can help you in your work practices, what would they be?

- Opinions about the prototype
  - What do you think about the relationship between the items and the social media analyses?
  - Do you think social media analyses can be a kind of collateral information you will use?
  - Would you be willing to adopt this tool if possible? If not, why?
  - What do you think about the time frame of the visualization?
  - What do you think about being able to see patient social media information prior to when they began treatment? (from ethical perspectives)
  - Possible scenarios where this tool can be the most helpful (type of patients, the specific moment in their interactions, etc)
  - Possible scenarios where this tool can be the least helpful?
  - Do you think this could have an impact on the therapeutic relationship? If so, how?

- Burdens/barriers
  - Do you see any barriers which may prevent you from using this prototype in your everyday practice?

        - job constraints - do you think this prototype could fit into your work practice?
        - time constraints - how much time do you think you can invest in using this prototype in your everyday activities
    - Do you see any possibilities where patients may feel uncomfortable using this prototype in their treatment?
        - Do you think the data collection process is transparent enough to make patients comfortable?
        - Do you think the prototype is transparent enough to make patients comfortable?

- Concerns and thoughts
    - Do you have any concerns/thoughts regarding using social media analyses in your current work practices?
    - Do you have any ideas which may assuage some of your concerns?

- Potential impact on the therapeutic relationship
    - Do you think this could have an impact on the therapeutic relationship? If so, how?
- Knowledge/skills
    - Are you familiar with the concept of social media analysis?
- Beliefs about consequences
    - What do you think will happen if you use this prototype?
        - What will happen to patients? colleagues? organizations?
    - What are the costs of using this prototype? What are the costs of the consequences of x?
    - Do the benefits of using this prototype outweigh the costs?
- Motivation and goals
    - How much do you want to use this prototype?
    - How much do you feel you need to use this prototype?
    - Are there other things you want to do or achieve that might interfere with this prototype?
- Environmental context and resources
    - To what extent do physical or resource factors facilitate or hinder using this prototype?
    - Are there competing tasks and time constraints?
- Collaboration
    - Do you think this tool can facilitate/hinder collaboration?
