# Supplementary material for: Clinician Perspectives on Using Computational Mental Health Insights From Patients’ Social Media Activities: Design and Qualitative Evaluation of a Prototype
Source: JMIR Ment Health. 2021 Nov 16;8(11):e25455. doi: 10.2196/25455 (PMC8663497; doi:10.2196/25455)
Supplement: Multimedia Appendix 3 [file mental_v8i11e25455_app3.pdf]

Themes	Codes	Description	Example
**Current Work Practice**			
	Technology use during consultations	Clinician explains how they use their technology (mainly desktop) during their consultations	"I usually just I don't like to write things down or while I'm talking to them, because I want to show them that I'm focused. I usually just try to remember it. If there's anything particular that's very important, I'll write one or two things down."
	Collaboration	Clinician describes how they collaborate with other types of clinicians	"There's nothing that's structured. So for example, we work a lot in teams, right? So if I'm doing medication management, I'm seeing the patient once a month. But another team member is seeing them for therapy once a week. That person, who's seeing them once a week, would maybe relay a message to me that they're concerned about symptoms worsening or the patient mentioned they might be running out of medications or something like that. And then I would probably call them and reach out and say, "How are you doing? How are your medications? Are you running out? Do you need a refill? Do you want to come see me earlier?" Things like that. So that would be just based on team work clinicians alerting each other to changes with the patients that they share."
	Prior experience with patient social media	Clinician explains their prior experience when their patients brought up their social media content during their consultations	"Yeah. So, sometimes people will say, "Oh, let me show you this text." Or "Let me show you this Snapchat." Or "Let me show you this post." It certainly doesn't happen all the time, but occasionally... Or if someone is in the middle of a breakup, they want to show me something somebody said or posted or wrote to them. So, it's usually kind of in relation to something from their social lives."
	Compatability of the prototype	Clinician describes whether the prototype would be compatible with their current work practices	"I just think it would take practice to do it in such a way that the patient also doesn't feel neglected. Because that's something that's hard in all of medicine right now, is it's becoming a lot more electronic, anytime you go to see any doctor, they'll be on the computer while talking to you, and you will have to strike that balance. It's something that will take practice."
**Potential use cases**			
	Collateral information	Clinician mentions the prototype has a vaule as collateral information or the similarity between the prototype and other types of colleteral information (eg, a report from family)	"As long as they know that this is something that they've done, they know that I'll be checking it; yes, I think it could be really useful data to bring up during the session because it provides you with some comparisons to what you're saying."
	Collaborative agenda setting	Clinician describes how they would like to review the prototype together and to use conversation surrounding the prototype as agenda for the consultation	"I think that, you know, some providers might use it in like a gotcha way, but if we have the patient in the office and we asked her questions, and me say like, "Okay, let's spend like five minutes and let's go through your data together." And we look at the graphs together. And then point out like, oh, it looks like this period, you were posting a lot at nighttime, what was going on then? And you just use it as a way to further explore if something didn't come up in the session."
	Tracking symptoms	Clinician sees value in tracking symptoms using the prototype	"I think like it's definitely of big help, because when patients improve later on, they might not remember facts correctly. They might sometimes say that like, "I don't know, this thing never happened. I have always been doing good." A little bit of denial sets in once you get better, but for things that you have posted on social media, as you were saying, there remains a permanent record, even if you just wrote it in the moment or even if you just wrote it out of impulse, I mean, sometimes that can be explained, like this person was just writing this on a social media out of impulse and he has a history of doing things as a part of impulsive behavior."
	Specific conditions	Clinician explains certain types of patients (eg, bipolar disorder patients) can potentially benefit from the prototype	"So I guess maybe in a bipolar patient that would be more helpful. If they're saying, "Oh yeah, I'm sleeping well" but they're posting throughout the whole night, then you could see that their sleep patterns are off."
**Concerns**			
	Liability	Clinician express their concerns related to liability or legal issues	"I don't know legally, how that works. What if you can't reach them. Do you have a legal responsibility then to send someone to their house or whatever. "

	Credibility	Clincian questions the credibility of mental health insights from the prototype	"And how does that algorithm delineate that certain posts are more likely to be related to depression versus others? Like if somebody had just posted they're listening to some dark music, would it automatically pick that they're suffering from low mood, that's why they're ... Because sometimes people just write that on their Facebook post, they're listening to this, and, like, that's a part of dark music, or in general, sad songs."
	Systematic support	Clinician states they would need instituitional supports to be able to use this prototype in their daily practices	"I think it's also... for some reason I found it a little complex so that I need to... let's say in clinical if I see a patient, a new patient and then I can tell the patient and the parents that, "Hey, by the way, I have this software or the app that we could use, that can also help me understand how depressed you are or so that... but then you have to give this software, i.e. me, an access to get information from your social media account." So that's a little, I don't know how I would explain to my patient so I find it a little complex but then, I thought the system was easy to use. I think the usability probably is simple, you could say that at the beginning explaining to the patients."
	Obsever effect	Clinician foresees that the prototype would impact patients' behaviors on social media, which may make the prototype less useful	"The concern that everything that they do or even if they're being monitored, big brother's watching and even though consents are signed, I mean, paranoia is what, an irrational fear and they're very vulnerable. So, it can go the other way too."
	Patient characteristics	Clinician envisions cases where the prototype would be less helpful based on certain patient characteristics (eg, their conditions and symptoms)	"Well, I think the less helpful would be you know, patients with more access to pathology or like things like Borderline Personality Disorder, if you suspect they're doing it more for attention. And if you know from their history, like their actual intent when they make statements like that is not always that high. So that's situations where it could create a lot of red flags for you. But then like not lead to anything necessarily productive."
**Design suggsetions**			
	Additional information	Clincian expresses their need for addtional types of information that are not in the prototype	"Probably like homicidal thoughts in a violent patient or a psychotic patient. Like any paranoid or psychotic type thoughts, violent... I mean you could also track like how often they're posting about substance, alcohol, you know like that kind of stuff for this."
	Trends	Clinician expresses their need for understanding trends over time in the prototype	"then see it like sort of graphically. I could even just toggle between this raw data view and and graphically track, with a trendline embedded. Or just add trend line kind of thing."
	Concise view	Clinician suggests that more concise information would be better (eg, summary view)	"if there was some sort of summary statistic for like a week or over the course of a month, that I could see or like graphically represented."
	Correlation	Clinician states that they would like to learn/see the correlation between different categories (eg, the correlation between depression and insomnia)	"it seems like the timing is correlating with the less nighttime social media posting is correlating with the less depression and less suicidal thoughts. I'd want to understand the connection with that."
	General FB use	Clinician mentions they would like to learn the general Facebook use of the patient (eg, the total number of posts during the period)	"Is there any way to know how many times they log into Facebook? Why are we seeing something is not lining up, it's because they're not using it or whatever."
	Actual post	Clinician asks whether they could read the actual post content or states they would like to read it if possible	"I would really think it could be helpful on the suicide one if you could give us examples of the actual statements that you pulled from the social media. For example, if the statement is kill me now, as opposed to, if somebody doesn't call me in five minutes, I'm jumping off this bridge. I think there's a very different connotation and severity to that. I think being able to see the actual things you pulled from the suicidal stuff could be helpful to the clinician in the moment. "
